# Pauses in Cholinergic Interneuron Activity Are Driven by Excitatory Input and Delayed Rectification, with Dopamine Modulation

**DOI:** 10.1016/j.neuron.2018.04.027

**Published:** 2018-06-06

**Authors:** Yan-Feng Zhang, John N.J. Reynolds, Stephanie J. Cragg

**Affiliations:** 1 Department of Physiology, Anatomy and Genetics, University of Oxford, Oxford OX1 3PT, UK; 2 Oxford Parkinson’s Disease Centre, Oxford OX1 3PT, UK; 3 Department of Anatomy and the Brain Health Research Centre, Brain Research New Zealand, University of Otago, Dunedin 9054, NZ

**Keywords:** striatum, cholinergic interneuron, basal ganglia, pause response, delayed rectification, excitatory input, corticostriatal, thalamostriatal, nigrostriatal, dopamine

## Abstract

Cholinergic interneurons (ChIs) of the striatum pause their firing in response to salient stimuli and conditioned stimuli after learning. Several different mechanisms for pause generation have been proposed, but a unifying basis has not previously emerged. Here, using *in vivo* and *ex vivo* recordings in rat and mouse brain and a computational model, we show that ChI pauses are driven by withdrawal of excitatory inputs to striatum and result from a delayed rectifier potassium current (I_Kr_) in concert with local neuromodulation. The I_Kr_ is sensitive to K_v_7.2/7.3 blocker XE-991 and enables ChIs to report changes in input, to pause on excitatory input recession, and to scale pauses with input strength, in keeping with pause acquisition during learning. We also show that although dopamine can hyperpolarize ChIs directly, its augmentation of pauses is best explained by strengthening excitatory inputs. These findings provide a basis to understand pause generation in striatal ChIs.

**Video Abstract:**

## Highlights

• Withdrawal of excitatory input to striatum induces pauses in ChIs
• Delayed rectification by a K_v_7-mediated potassium current (I_Kr_) underlies pauses
• Synapse weighting rather than direct hyperpolarization by dopamine promotes pauses
• ChIs are faster to respond to excitatory input than striatal projection neurons

## Teaser

Zhang et al. reveal how synchronized pause responses in striatal cholinergic interneurons are driven, through the response of a delayed rectifier current to withdrawal of excitatory inputs, in conjunction with dopamine acting to potentiate the pause during learning.

## Introduction

Cholinergic interneurons (ChIs) constitute only 1%–2% of striatal neurons but are emerging as key players in action selection, reinforcement, associative learning, and behavioral flexibility (Aoki et al., 2015, Bertran-Gonzalez et al., 2013, Bradfield et al., 2013, Joshua et al., 2008, Matamales et al., 2016, Maurice et al., 2015, Morris et al., 2004, Okada et al., 2014). ChIs fire tonically at 3–10 Hz (Apicella et al., 2009, Kimura et al., 1984, Wilson et al., 1990) but also demonstrate phasic responses consisting of short pauses flanked by preceding and/or “rebound” phases of increased ChI activity (Aosaki et al., 1994a, Aosaki et al., 1994b, Apicella, 2007, Apicella et al., 2011, Kimura et al., 1984, Ravel et al., 1999). These phasic changes occur in response to salient or reward prediction-related stimuli after conditioning, implicating them in learning and action selection. Interest in ChI pauses has been reinforced by the finding that they coincide with phasic activity in midbrain dopamine (DA) neurons (Joshua et al., 2008, Morris et al., 2004).

The mechanisms responsible for generating pauses and enabling their acquisition during learning have been long investigated but remain incompletely reconciled. Different approaches have suggested diverse mechanisms that include an I_h_ current-dependent after-hyperpolarization (AHP) following action potentials or subthreshold excitation, plasticity of excitatory inputs, GABA input (in ventral striatum), and DA D_2_-receptor-mediated inhibition (Aosaki et al., 1994a, Brown et al., 2012, Ding et al., 2010, Kharkwal et al., 2016, Oswald et al., 2009, Reynolds et al., 2004, Reynolds and Wickens, 2004, Suzuki et al., 2001, Wilson, 2005, Zhang and Cragg, 2017). The DA dependence of the pause is a hypothesis that has gained particular traction. Depletion of DA *in vivo* limits pause development in response to conditioned stimuli (Aosaki et al., 1994a), and *ex vivo* stimulation of DA release in slices acutely induces a D_2_-dependent pause in ChI firing (Ding et al., 2010, Kharkwal et al., 2016, Straub et al., 2014, Wieland et al., 2014). And yet, when ChIs and DA neurons are recorded in the same tasks *in vivo*, the ChI pause response does not show proportionality to DA neuron firing rate in either latency or amplitude (Joshua et al., 2008, Morris et al., 2004), suggesting that acute activation of D_2_ receptors plays a limited role in pause generation *in vivo*. An underlying basis for pause expression that accommodates previous observations, including a role for DA, has, until now, remained undefined. Here, by exploring pauses *in vivo*, *ex vivo*, and *in silico*, we reveal a mechanism for ChI pause expression that reconciles and revises our understanding of the different contributing factors. We show that pauses are driven during recession from excitatory input by a delayed rectifier current and with regulation of excitatory synapse strength by neuromodulators serving to modulate pause acquisition.

## Results

### ChIs *In Vivo* Respond to Changing Excitatory Input

**Figure 1:**
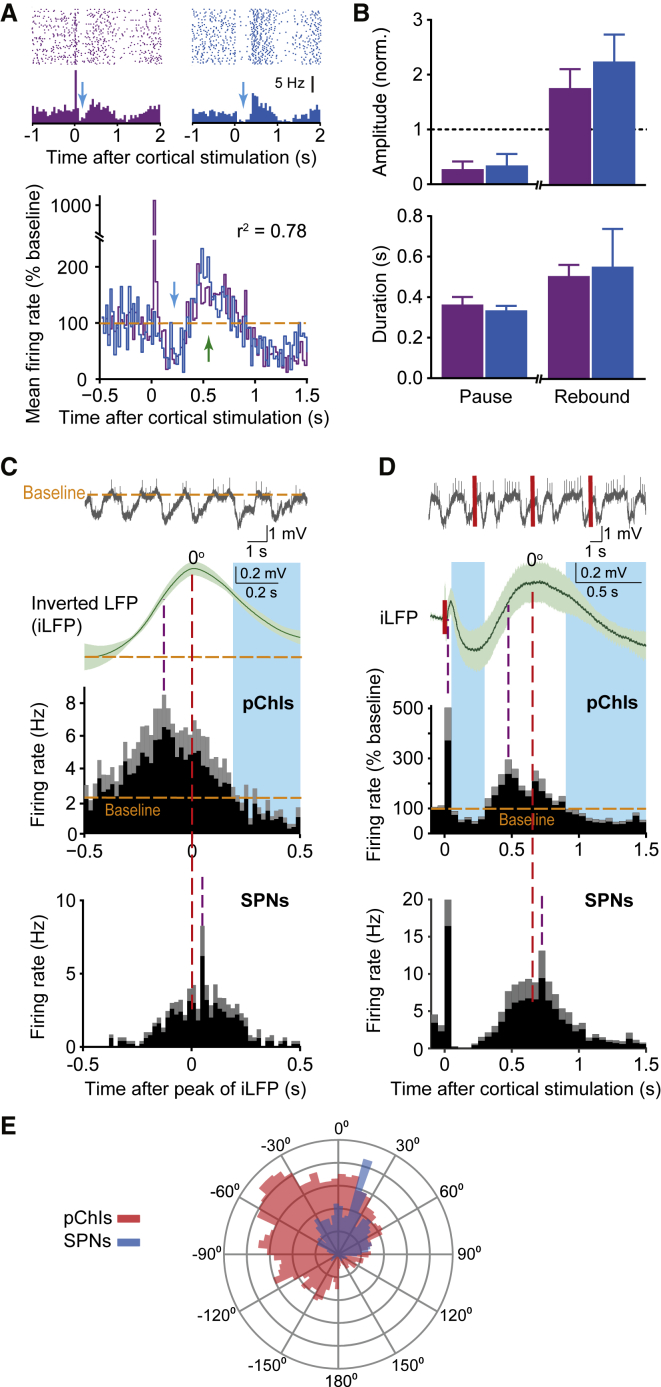
*In Vivo* Firing Rate of pChIs Reflects Changes in Excitatory Input, with Pauses Accompanying Withdrawal of Excitation (A) Firing of pChIs with (left, purple) or without (right, blue) short-latency-evoked action potentials before a pause. Top: example; bottom: average, pause (blue arrow), rebound (green arrow). Firing rate correlation, *r*^2^ = 0.78, p < 0.001 (n = 4–5). (B) Mean ± SEM for amplitude (top) and duration (bottom) of pause and rebound. (C and D) Top: example striatal LFP; middle top: mean ± SEM of inverted LFP (iLFP); middle: mean firing rate (black) ± SEM (gray) in pChIs (n = 5, n = 9); bottom: mean firing rate (black) ± SEM (gray) in SPNs (n = 5) aligned to maximum of spontaneous iLFP (dashed red line) (C) or contralateral cortical stimulation (0.2 Hz; solid red lines) (D). Purple dashed line, firing rate maxima; shaded blue, pChI firing rate below baseline, a “pause.” (E) Phase plot of firing rates for ChIs and SPNs. Data were extracted during slow LFP oscillation in (D).

We explored ChI pause generation *in vivo* by recording single-unit activity in putative ChIs (pChIs) in urethane-anaesthetized rats (Figures S1A and S1B). We first corroborated previous observations that an evoked ChI pause does not require action potentials (Aosaki et al., 1994b) evoked by cortical stimulation. While some neurons demonstrated a short-latency increase in action potential firing rate (<20 ms), others did not, yet both types of responding neurons exhibited pause and rebound responses (Figure 1A). Pause and rebound responses did not differ in amplitude or duration between neurons whether or not an action potential was evoked (Figure 1B).

However, during spontaneous slow-wave activity (Figure 1C) or after stimulation of contralateral motor cortex (Figure 1D), ChI firing rate co-varied with an inverted function of the striatal local field potential (inverted, iLFP), a proxy of excitatory input (Mahon et al., 2001, Ryan et al., 1986). ChI firing rate increased to maximum during the ascending phase of the iLFP (prior to iLFP maximum) but decreased below baseline rate, i.e., “paused,” during the receding phase of the iLFP despite the iLFP value exceeding baseline. Similar relationships can be observed in other datasets (Berke et al., 2004, Reynolds et al., 2004, Ryan et al., 1986, Schulz et al., 2011, Sharott et al., 2012). By contrast, the firing rate of identified striatal projection neurons (SPNs) had a later onset and peak of elevated activity, with firing rate peaking during the receding iLFP and lagging behind pChIs by 60° (Figures 1C–1E). These responses indicate that ChIs are early responders to afferent input, preceding SPN responses. They suggest that ChIs *in vivo* respond to changing excitatory input and pause in response to receding excitatory input, with pause duration curtailed by subsequent excitatory input.

### Striatal ChIs *Ex Vivo* Pause in Response to Receding Excitatory Input

**Figure 2:**
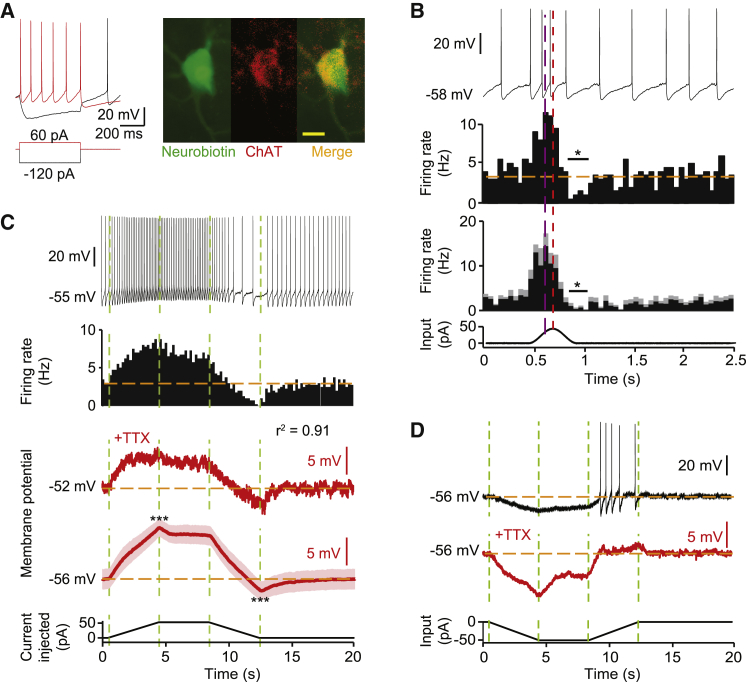
ChI Firing *Ex Vivo* Rate Reflects Changes to Excitatory Input and Pauses Are Driven by Withdrawal of Excitation (A) Characteristic ChI physiology. Immunocytochemical co-labeling: neurobiotin fill; ChAT-immunoreactivity (scale bar, 20 μm). (B) Example sweep, example firing rate histogram (20 sweeps), and mean firing rate histogram ± SEM (n = 6) of ChI response to a sine-wave current. Highest firing rate (purple dashed line), input current maximum (red dashed line), reduced firing rate versus baseline, ^∗^p < 0.05, t test. (C and D) Responses to trapezoid current injections for depolarizing (C) and hyperpolarizing (D) input. (C) Top to bottom: example sweep, example firing rate histogram (20 sweeps), and representative and mean membrane potential ± SEM in presence of TTX 1 μM (red). Correlation, firing rate and membrane potential, *r*^2^ = 0.91, 100 bins. ^∗∗∗^p < 0.001, paired t tests for maximum versus plateau and minimum versus baseline (n = 10). (D) Example sweep plus membrane potential in presence of TTX (red).

To test directly whether ChIs track changes in input and whether a decay of excitatory input is sufficient to pause firing, we tested the effect of manipulating input to ChIs in *ex vivo* slice preparations. ChIs recorded using current clamp in mouse and rat striatal slices (Figure 2A; Figure S1C) were injected with current mimicking excitatory input fluctuation *in vivo* seen in the iLFP (Figure 2B; Figure S1C). ChI firing rate peaked during the input ascending phase and was minimal during the input decay phase when it fell transiently below baseline (Figure 2B; Figure S1C), resembling 200 ms pauses seen *in vivo* in monkeys (Aosaki et al., 1994b). We temporally separated the ascending and descending components of current injection to resolve how different components of input govern ChI activity (Figure 2C; Figure S1D). Changes in ChI firing rates resembled “overshoot” and “undershoot” responses: changes from baseline were maximal during changes in input and lessened during plateau levels of input (Figure 2C). SPNs, by contrast, did not have overshoot/undershoot responses even at similar membrane potentials (Figure S2).

In ChIs, changes in underlying membrane potential were a proxy for pauses in firing: membrane potential responses to current ramps in the presence of TTX were correlated with firing rate without TTX (Figure 2C; Figure S1D), and cessation of hyperpolarization and pauses were aligned across experiments (Figures 2B and 2C). Furthermore, the size of ChI overshoot/undershoot in membrane potential scaled with amplitude of input current (Figure S3A) and with the rate of current withdrawal (Figure S3B) and also occurred in response to hyperpolarizing current (Figure 2D). In addition, during recordings of firing activity, we applied a small, negative current calculated to generate a hyperpolarization equivalent to the undershoot observed following excitatory input and found that this current generates a short pause in ChI firing (Figure S4A). Taken together, these findings indicate that ChI firing rate reflects changes in the net amplitude of input.

### Pauses Due to I_Kr_

**Figure 3:**
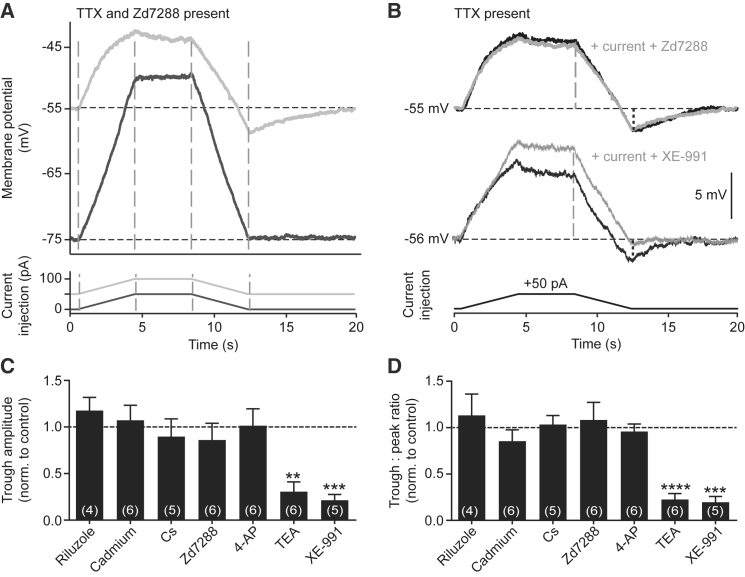
I_Kr_ Underlies Hyperpolarization Induced by Excitatory Input Withdrawal in ChIs (A) Representative membrane potential in presence of TTX (1 μM) and I_h_ blocker Zd7288 (50 μM) without (dark gray) and with (light gray) resting membrane potential (RMP) restored to −55 mV (n = 6) during trapezoid current injections. (B) Representative membrane potential in presence of TTX (black) and either (gray) I_h_ blocker Zd7288 or Kv7.2/7.3 blocker XE-991 (100 μM, n = 6). RMP was held at pre-drug condition. (C and D) Mean ± SEM of amplitude of hyperpolarization below RMP (C) or ratio of trough:peak (black versus gray vertical dashed lines in B) (D), normalized to control. Riluzole, 100 μM; cadmium, 200 μM; CsCl, 2 mM; Zd7288, 50 μM; 4-AP, 1 mM; TEA, 20 mM. n = 4–6. Typical traces shown in Figure S3. ^∗∗∗^p < 0.001, one-sample t test versus control.

We pursued the ionic mechanism responsible for the overshoot/undershoot in membrane potential in mouse ChIs in the presence of TTX. A blocker of the hyperpolarization-activated cyclic nucleotide-gated (HCN) current I_h_, Zd7288, eliminated overshoot/undershoots (Figure 3A), but this was due to the direct hyperpolarizing effects of I_h_ block, because when resting membrane potential was restored by current injection, the overshoot/undershoot responses were restored (Figures 3A and 3B). Similar outcomes were noted with CsCl, another blocker of I_h_ (and other K^+^ channels) (Figures 3C and 3D; Figure S3C). These data indicate that the hyperpolarization responses in membrane potential that underlie pauses are mediated by a voltage-dependent mechanism. Membrane potential is I_h_ dependent as expected, but the I_h_ current does not mediate the pause.

The hyperpolarization was not modified by the persistent Na^+^ channel blocker riluzole, the broad-spectrum Ca^2+^ channel blocker cadmium, the fast A-type K^+^ channel (I_A_) blocker 4-AP (Figures 3C and 3D; Figure S3C), D_2_ receptor antagonist L-741626, or antagonists for GABA_A_ receptors or nicotinic ACh receptors (Figures S3C and S3D) (but see later for DA effects). By contrast, the broad-spectrum K^+^ channel blocker TEA attenuated the undershoot (Figures 3C and 3D; Figure S3C). Of the candidate K^+^ channels not blocked by other agents, TEA blocks non-inactivating delayed rectifier K^+^ currents (I_Kr_), which constitute the slow or persistent component of I_A_ in ChIs (Song et al., 1998). To identify which Kv channel mediates this I_Kr_, we screened, in pilot experiments, a range of blockers of candidate Kv channels (Kv1, -2, -4, -7, and -11) for their ability to block the undershoot. We identified that the Kv7.2/7.3 antagonist XE-991 (Petrovic et al., 2012) prevented the undershoot (Figures 3B–3D), thereby identifying Kv7.2/3 channels as mediators of the I_Kr_ responsible for pause generation in ChIs.

### I_Kr_ Can Provide the Hyperpolarization Response in a Computational Model

**Figure 4:**
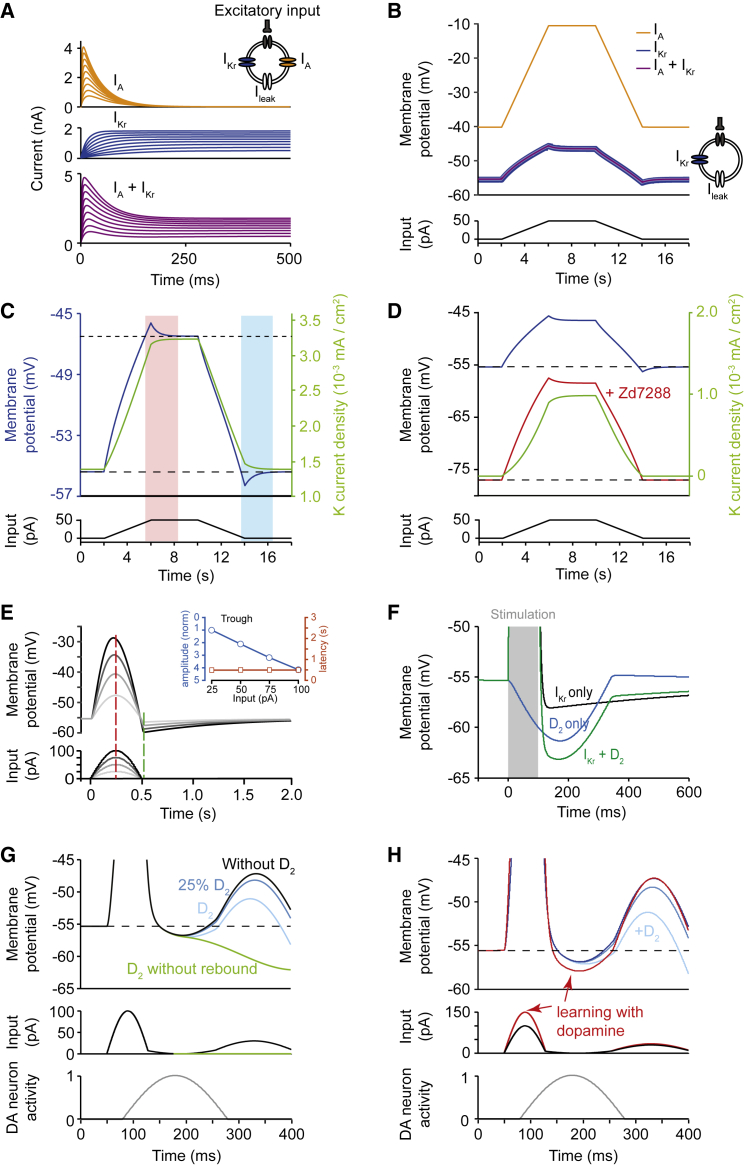
I_Kr_ Underlies Hyperpolarization and Interacts with Dopamine in a Model ChI (A) Current-time responses of conductances, 20 mV steps from −100 mV; inset: single-compartment neuron model. (B) Membrane potential response to synaptic input (current), with I_leak_ and I_Kr_ and/or I_A_. (C) I_Kr_ current density (green) and membrane potential (dark blue) showing overshoot (pink area) and undershoot (blue area). (D) Undershoot lost (red) at membrane potential −80 mV is restored with depolarization to normal RMP (blue). (E) Response to short sine-wave input. Maximum current injection (red dash); undershoot (green dash). Inset: trough amplitude (blue), but not latency (orange), scales with amplitude of current injected. (F) Effect of D_2_ current and I_Kr_ after separate and combined activation after a stimulation (gray area) starting at time zero. (G and H) Membrane potential response (top) to input to ChIs (middle) flanking a DA neuron burst (bottom) with 100% (light blue) or 25% (dark blue) of D_2_ currents identified in Straub et al. (2014) before (G) and after (H) enhanced excitatory input following learning (Suzuki et al., 2001). In (G), response without rebound (green). I_leak_ present throughout.

We constructed a computational model to test whether the I_Kr_ is sufficient to drive pauses and to explore how the I_Kr_ drive can govern aspects of pauses observed *in vivo* and *ex vivo*. A model cell containing an I_A_ (fast component) and I_Kr_ (Figure 4A) responded to a ramping excitatory input (depolarizing current) with overshoot and undershoot of membrane potential (Figure 4B) as seen *in vivo* and *ex vivo* (see Figures 1, 2, and 3). Furthermore, these responses were generated by I_Kr_ alone, but not by I_A_ alone (Figure 4B). The I_Kr_ dependence can be rationalized from the current density: the voltage dependence and slow time constant of the I_Kr_ (see Figure 4A) result in an outward current that reaches maximum/minimum later than those of the depolarizing input (Figure 4C). The outward delayed rectification by the lagging I_Kr_ current thus permits a corresponding overshoot/undershoot in membrane potential before reaching steady state (Figure 4C). A pause will occur when the slowly changing I_Kr_ exceeds the receding excitatory input.

The I_Kr_-containing model cell also accounted for other attributes of pauses: the post-excitation undershoots in membrane potential were appropriately voltage dependent (Figure 4D, as seen in Figure 3); troughs scaled with input (depolarizing, Figure 4E; hyperpolarizing, Figure S4B; as seen in Figure S3A) as seen during acquisition of pauses *in vivo* during learning (Aosaki et al., 1994b) when excitatory input is enhanced; and trough onset latency and peak timing were constant as can be noted for pauses during learning *in vivo* (Aosaki et al., 1994b, Zhang and Cragg, 2017).

Furthermore, we used the model to rationalize the different described effects of DA to promote pauses acutely *ex vivo* but progressively during learning *in vivo*. We incorporated an acute DA D_2_-receptor-dependent hyperpolarizing current (“D_2_ current”) with amplitude and latency quantified from ChIs following optogenetic activation of DA axons (Straub et al., 2014) (Figure 4F, blue), in combination with different activity in ChIs. When we simulated DA release in response to optogenetic activation of ChIs or striatal electrical field stimulation (Ding et al., 2010, Kharkwal et al., 2016, Threlfell et al., 2012), the resulting D_2_ current summated with the I_Kr_ activated by ChI excitation to promote the hyperpolarizing undershoot in ChI membrane potential (Figure 4F). When we modeled activation of DA neurons to occur later, coincident with a pause in ChIs as occurs *in vivo* (Joshua et al., 2008, Morris et al., 2004), the D_2_ current promoted ChI hyperpolarization, but at a later time point, which prolonged the pause compared to I_Kr_ alone (Figure 4G, D_2_ without rebound). But when we additionally incorporated ChI rebound activity seen *in vivo*, the D_2_ current had very limited effects on hyperpolarization amplitude or duration (Figure 4G). The trough was I_Kr_ dominated, although the D_2_ current could decrease the amplitude of rebound. To validate the finding that these different contributors, I_Kr_ and D_2_, have distinct timing and efficacy on pausing, we recorded activity in ChIs *ex vivo* and activated DA axons at the beginning of a ChI pause. Brief optogenetic activation of DA axons released DA and briefly inhibited action potentials in ChIs (Figure S4C) as shown previously (Straub et al., 2014). In agreement with our simulations, the DA-dependent inhibition coincided with the end of the excitation-induced pause in ChI activity and the beginning of rebound activity (Figure S4D), suggesting that DA does not acutely modify the pause during ongoing excitatory input.

By contrast, when we modeled a different effect of DA, namely a potentiation of excitatory input to ChIs that has been proposed during learning (Fino et al., 2008, Reynolds et al., 2004, Suzuki et al., 2001), a greater I_kr_ was activated, which consequently enhanced hyperpolarization on excitatory input withdrawal (Figure 4H). These findings suggest that the indirect potentiation by DA of synaptic input and I_Kr_, but not D_2_-mediated hyperpolarization, promotes the amplitude of pauses *in vivo*.

## Discussion

We show that ChIs report fluctuations in their inputs and pause when excitatory input recedes. These characteristics are mediated by the slow, non-inactivating, delayed rectifier current I_Kr_ carried by Kv7.2/7.3 channels. We reconcile these findings with the modulation of pause expression by DA. DA can acutely inhibit ChI excitability and also weight excitatory inputs, but effects on synaptic weighting are better placed to promote pause expression *in vivo*.

### Withdrawal of Excitatory Input Induces Pauses

ChIs in dorsal striatum receive excitatory glutamatergic input from cortex and thalamus (Guo et al., 2015, Lapper and Bolam, 1992). We show that ChIs have a differentiator-like response to input, reflecting escalating or receding activity. Pauses in firing *in vivo* are promoted not by a single AHP induced by an action potential, but rather by the receding of activity in the excitatory network. This mechanism does not apply to SPNs. In ChIs, this outcome is mediated by the delayed rectification properties of the slow, non-inactivating I_Kr_. This current is also called the slow A current (Song et al., 1998) and is carried here by Kv7.2/7.3, a channel that can delay action potentials in tonically firing neurons (Brown and Passmore, 2009). Other intrinsic currents have previously been proposed to mediate the ChI pause response, notably transient inactivation of the I_h_ current (Bennett et al., 2000, Oswald et al., 2009) since I_h_ inhibition diminishes the size of AHPs in brain slices. However, we show that inhibition of I_h_ leads to a hyperpolarization, which limits dynamic activation of the I_Kr_. Thus, the I_h_ current does not mediate the pause but rather plays a permissive role in pause expression by maintaining sufficient depolarization for activation of the I_Kr_. The I_Kr_ has a sufficiently slow voltage dependence of activation and inactivation to give rise to highest and lowest ChI firing rates during increases and decreases in net excitation, with hyperpolarizing undershoots that underlie pauses being more strongly driven by faster withdrawal from stronger excitation. Additional currents that contribute to hyperpolarization, e.g., KIR (Wilson, 2005), regulate inter-spike interval of spontaneous action potentials but do not account for the duration of conditioned pauses induced by inputs.

These results demonstrate, for the first time, that ChIs are entrained by the fluctuation, but not the absolute value, of the synaptic input. In order to pause, ChIs do not need the large level of inputs required to drive an action potential for a prolonged AHP but rather need only a smaller change of a few picoamps to change the membrane potential sufficiently to activate I_Kr_. Consequently, ChIs distributed sparsely in the striatum could therefore be effectively synchronized by small net changes in network activity, arising from either recession from excitation or an incoming inhibitory input.

Furthermore, by comparing the firing of ChIs and SPNs to the phase of excitatory input, we confirmed that ChIs provide an early readout of striatal input that precedes changes in SPNs firing. This timing could be critical to local signal processing and to striatal plasticity, including spike-timing-dependent plasticity. Changes in inputs to ChIs in pathological states could, in turn, have significant implications for timings within striatal microcircuits, e.g., due to modified corticostriatal connectivity in attention deficit and hyperactivity disorder (ADHD) (Bush, 2010) or degeneration of thalamostriatal inputs in Parkinson’s disease (Halliday, 2009, Smith et al., 2014).

### Interactions with Dopamine and Learning

Our data give a framework to reconcile different observations relating to the role of DA in pauses *ex vivo* and *in vivo*. In a model cell with an I_Kr_, D_2_ receptor activation without subsequent synaptic inputs can generate an acute and prolonged hyperpolarization that matches data in slices (Ding et al., 2010, Kharkwal et al., 2016, Straub et al., 2014). However, when DA neuron activity and D_2_ currents were modeled *in silico* or induced *ex vivo* to coincide with a ChI pause as occurs *in vivo*, the D_2_ current occurred too slowly to potentiate the coincident ChI pause, which was instead dominated by the faster I_Kr_. Furthermore, on subsequent excitatory input after a ChI pause, the effects of the D_2_ current are offset by the rebound activity arising in ChIs.

These findings suggest that the efficacy of DA *in vivo* to enhance pauses does not lie in its acute effects. This deduction fits with observations *in vivo* that, during learning, the acquisition of conditioned pauses does not necessarily manifest as increased duration, but rather increased amplitude (Aosaki et al., 1994b), which the D_2_ current would appear too latent to mediate. Moreover, it also fits with observations that, after learning, pause amplitude does not scale with concurrent DA neuron firing rate: the probability of conditioned reward or reward prediction errors are signified by a positive monotonic relationship in DA neuron firing rate (Joshua et al., 2008, Morris et al., 2004, Schultz et al., 1997), whereas the amplitude of the coincident ChI pause is invariant (Joshua et al., 2008, Morris et al., 2004).

An additional effect of DA is potentiation of excitatory inputs to ChIs (Bonsi et al., 2004, Fino et al., 2008, Reynolds et al., 2004, Suzuki et al., 2001). We show that the documented potentiation of excitatory inputs by DA is sufficient to promote the activation of I_Kr_-mediated hyperpolarization on input recession. When promoted, the I_Kr_, although small in size, should be sufficient to delay the timing of action potentials against the weak sodium current that pulls the ChI to their threshold (Wilson, 2005). These data indicate that it is the action of DA to strengthen excitatory inputs to ChIs that will enhance pause acquisition and amplitude. One additional means through which DA might gate the pause could be when pause responses are well developed, when synchronized increased activity in a population of ChI activity occurs prior to a pause (Joshua et al., 2008, Morris et al., 2004) and can drive DA release (Cachope et al., 2012, Ding et al., 2010, Threlfell et al., 2012). In that case, a D_2_-mediated current might have appropriate timing to contribute to ChI pause amplitude, but its contribution remains unknown.

We also note that our model suggests that acute DA could potentially act to modulate the amplitude of so-called rebound activity in ChIs. However, rebound amplitude correlates positively rather than negatively with expected reward value (Apicella et al., 2011), suggesting that DA availability *in vivo* does not govern rebound amplitude via D_2_ currents. Other inputs have been proposed to influence rebound, which may be a separate phase of activity unrelated to pause amplitude, including a long-latency intralaminar thalamic input (Matsumoto et al., 2001, Schulz et al., 2011) and a D_1_ current (Wieland et al., 2014).

The I_Kr_ mechanism that we identify could explain most known observations of the pause response in behaving monkeys, where alternative theories for pause generation are insufficient. The I_Kr_ mechanism will enable ChIs to pause in response to small fluctuations in input, e.g., a decay of excitation as small as 25 pA (Figure S3A), which in turn will enable ChIs across hemispheres to pause in synchrony in response to small fluctuations in network-level activity (Aosaki et al., 1995). Prior spike activity, i.e., initial excitation, will not be necessary to pause ChIs (Aosaki et al., 1994b). Shorter pauses in aversive compared to appetitive tasks may reflect that excitatory input fluctuation is faster in aversive tasks (Ravel et al., 2003). A mechanism driven by synaptic excitatory input, unlike one driven by more diffuse actions of striatal DA as a volume transmitter, will also easily be able to differentiate neighboring ChIs to respond to some, but not other, stimuli (Apicella et al., 1997). In turn, ChIs that do not receive excitatory input in a certain task will not be expected to pause even with extensive training (Aosaki et al., 1994b, Apicella et al., 2009, Ravel et al., 1999). The weakness of the I_Kr_ prior to learning can potentially explain why ChIs spike at a slower rate than baseline during the pause before animals are extensively trained (Aosaki et al., 1994b). In addition, the I_Kr_ mechanism could potentially explain why a second pause can follow the rebound (Apicella et al., 2011, Ravel et al., 2003, Ravel et al., 2006), whereas the second pause is not coincident with phasic activities of DA neurons.

In summary, we reveal that intrinsic properties of ChIs favor the generation of pauses in response to changes in input, including withdrawal of excitation, with an amplitude that varies with the strength of input and the timing of striatal neuromodulation. Our findings suggest that although DA can acutely promote ChI hyperpolarization, its effects on plasticity of excitatory inputs are more likely to mediate its contribution to pause expression. Through this basis, pauses will be driven acutely by strong excitatory or inhibitory inputs, e.g., excitatory thalamic inputs in response to unexpected sensory cues or rewards, and will also be acquired on the longer timescales involved in learning through potentiation of cortical/thalamic inputs by DA.

## STAR★Methods

### Key Resources Table

**Table:** 

REAGENT or RESOURCE	SOURCE	IDENTIFIER
**Antibodies**		

Anti-Choline Acetyltransferase Antibody (500 μL)	Millipore UK	AB144P
Streptavidin, Alexa Fluor 488 Conjugate 0.5 mL	Life Technologies	Cat. No. S-32354

**Chemicals, Peptides, and Recombinant Proteins**		

Bicuculline	Bio-Techne (R&D Systems)	Cat. No. 0130
Tetrodotoxin (TTX)	Bio-Techne (R&D Systems)	Cat. No. 1078
Riluzole hydrochloride	Tocris	Cat. No. 0768
Cadmium chloride	Sigma-Aldrich	439800-5G
Cesium chloride	Sigma-Aldrich	Cat. No. 289329-25G
Zd7288	Bio-Techne (R&D Systems)	Cat. No. 1000)
4-Aminopyridine (4-AP)	Bio-Techne (R&D Systems)	Cat. No. 0940
Tetraethylammonium chloride	Sigma-Aldrich	T2265-25G
L741626	Bio-Techne (R&D Systems)	Cat. No. 1003
Dihydro-β-erythroidine hydrobromide (DHβE)	Bio-Techne (R&D Systems)	Cat. No. 2349
NEUROBIOTIN	Vector	Cat. No. SP-1120
XE-991	Sigma-Aldrich	Cat. No. X2254-10MG
XE-991	Alomone Labs	Cat. No. X-100

**Experimental Models: Organisms/Strains**		

Long–Evans rats	Charles River	Strain Code: 006
C57BL6/J mice	Jackson Laboratory	Stock# 000664
DAT-cre mice	Jackson Laboratory	Stock# 020080
Ai32(RCL-ChR2(H134R)/EYFP) mice	Jackson Laboratory	Stock# 012569

**Software and Algorithms**		

pCLAMP	Molecular Devices	v.10.2
NEURON	Yale University	v.7.3
MATLAB	MathWorks	R2015a
Spike2	CED	v.6 or v.7
Scripts for NEURON ChI model	The authors; Deposited in Github.com	https://github.com/Yanfeng-Zhang/Pause-in-ChIs-Neuron-model

### Contact for Reagent and Resource Sharing

Further information and requests for resources and reagents should be directed to and will be fulfilled by the Lead Contact, Stephanie Cragg (stephanie.cragg@dpag.ox.ac.uk).

### Experimental Model and Subject Details

All *in vivo* procedures in this study were conducted in accordance with approvals granted by the University of Otago Animal Ethics Committee. Male Long–Evans rats (250–450 g) were group-housed and kept on a 12 hr light/dark cycle with *ad libitum* access to food and water.

Male adult (21-40 days) C57Bl6/J mice, DAT-cre;Ai32 mice (16-18 weeks), and Long-Evans rat pups (p15-20) were used for *ex vivo* experiments. After initial experiments in both rats and mice, we explored mechanisms regulating ChI activity in mouse only, which were selected over rats to enable complementary optogenetic manipulations in our mouse driver lines in subsequent experiments. DAT-Cre mice (B6.SJL-Slc6^a3tm1.1(cre)Bkmn^/J, JAX stock number 006660) were crossed with Ai32 mice (B6;129S-Gt(ROSA)26Sor^tm32(CAG-COP4∗H134R/EYFP)Hze^/J, JAX stock number 012569) to produce heterozygote DAT-Cre;Ai32 mice. Animals were group housed and maintained on a 12 hr light/dark cycle with *ad libitum* access to food and water. All procedures were performed in accordance with Animals (Scientific Procedures) Act 1986 (Amended 2012) with ethical approval from the University of Oxford, and under authority of a Project License granted by the UK Home Office.

### Method Details

#### *In Vivo* Recording

Long–Evans rats were anesthetized with urethane (1.4–1.9 g/kg, i.p.; Biolab), supplemented with additional urethane (0.2 g/kg) every 1-2 hr as required. All wounds and pressure points were infiltrated with bupivacaine (0.5%). Upon reaching surgical anesthesia, the head was fixed in a stereotaxic frame (Narishige, Japan). Core temperature was maintained at 35-36°C using a homeothermic blanket and monitored via a rectal probe (TR-100, Fine Science Tools). A round piece of skull overlying the right hemisphere (AP +2.0 mm and ML −1.6 mm to Bregma) was removed and a concentric stimulating electrode (Rhodes NEW-100X 10 mm, USA) implanted in the medial agranular motor cortex to a depth of 2.2 – 2.4 mm. Stimulating electrodes were connected to constant current electrical stimulators (Isolator-10, Axon Instruments). Stimulation pulses applied to the cortex were biphasic (0.1 Hz, 0.1 ms, 300 to 990 μA).

Extracellular single unit recordings were made using 5 – 15 MΩ micropipettes. Electrodes were filled with 1 M NaCl solution with 2% neurobiotin (SP1120, Vector). Recordings were made via either a headstage (model HS-2A) connected to an Axoprobe-1A microelectrode amplifier (Axon Instruments Inc California, USA), or a headstage (NL 100 Neurolog) connected to a preamp (NL104), an amplifier (NL106) and a filter (NL125). Signals were amplified and band-pass filtered (0.1 to 10,000 Hz). All waveform data were digitized at 50 kHz by an A-D interface (1401 Micro 2, CED, UK), and acquired using Spike2 software (v6 or v7, CED).

The micropipette was lowered through the striatum until a stable recording was obtained from a putative cholinergic interneuron (pChI) or striatal projection neuron (SPN). The pChIs included in this study showed a spontaneous tonic firing pattern (Figure S1) with long total spike durations (>1.1 ms) in the average waveforms. This distinguished them from SPNs, which exhibit a lower spike frequency (Stern et al., 1998) and fast spiking interneurons (FSIs), which have a shorter whole spike duration (Mallet et al., 2006). SPNs were identified by their broad average spike waveform (>1.1 ms) and slow spontaneous spike rate (<1 Hz). After recording, the neurons were actively filled with 2% neurobiotin by a juxtacellular filling protocol. The pChIs exhibited a regular firing pattern and their minimum inter-spike-intervals (ISIs) were greater than 20 ms, thus distinguishing them from the low threshold spiking interneurons (LTS neurons) which also fire tonically but with a bursty firing pattern and with ISIs less than 10 ms (Sharott et al., 2009). All data were analyzed offline with SPIKE2 and custom-written MATLAB (R2013b) scripts.

#### *Ex Vivo* Slice Recordings

For whole-cell patch-clamp and fast-scan cyclic voltammetry (FCV) in acute coronal slices, animals were anaesthetized with pentobarbital and transcardially perfused with ice-cold, high Mg^2+^ artificial cerebrospinal fluid (aCSF) containing in mM: 85 NaCl, 25 NaHCO_3_, 2.5 KCl, 1.25 NaH_2_PO_4_, 0.5 CaCl_2_, 7 MgCl_2_, 10 glucose, 65 sucrose. Brains were quickly removed over ice, blocked and 300 μm coronal slices were cut on a vibratome (Leica VT1200S) in the same solution. Slices between +1.5 to +0.5 mm from bregma containing caudate-putamen and nucleus accumbens were used. Slices recovered at 32°C for 30-40 min after dissection and were subsequently kept at room temperature. Slices were maintained and recorded in aCSF containing in mM: 130 NaCl, 25 NaHCO_3_, 2.5 KCl, 1.25 NaH_2_PO_4_, 2.5 CaCl_2_, 2 MgCl_2_, 10 glucose. The aCSF was saturated with 95% O_2_/ 5% CO_2;_ recordings were made at 32-33°C.

Whole cell patch clamp electrodes (3-7 MΩ) were filled with an intracellular solution containing in mM: 120 K-gluconate, 10 KCl, 10 HEPES, 4 MgATP, 0.3 NaGTP, 10 Na-phosphocreatine and 0.5% neurobiotin tracer. ChIs in the striatum were identified initially by their distinctive morphological features, i.e., large somas (>20 μm) and their characteristic electrophysiological properties, i.e., prominent I_h_, AHP and broad action potential. SPNs were identified by their medium size soma (10-20 µm), low RMP (≈ -85 mV), low input resistant (50 - 100 MΩ) and broad action potential. Recordings were made using a Multiclamp 700B amplifier and Digidata 1440A acquisition board with recordings digitized at 10-20 kHz. All data were acquired using Clampex and analyzed offline with Clampfit (pClamp10), and custom-written MATLAB (R2013b) scripts. Sine-wave currents injected were 25, 50, 75 and 100 pA, duration 500 ms. Trapezoid-shape current injection protocols used 4 s phases. There was minimal rundown of Kv7 I_Kr_ effects in ChIs, unlike rundown reported in other types of cells using different internal solutions and recording temperature (Simmons and Schneider, 1998). To mimic the I_Kr_ induced by a 50 pA sine wave at 2 Hz in Figure S4A, a negative current of −13.6 pA at a shape that is similar to the I_Kr_ hyperpolarization was injected to the ChIs. The amplitude (−13.6 pA) of the negative current was based on typical input resistance of ChIs (200 MΩ) and the hyperpolarization induced by an I_Kr_ (2.72 mV, Figure S3A). When recording D_2_ effects on ChIs evoked by optogenetic stimulation in Figure S4, bicuculline (10 μM) was applied to the bath to prevent GABA_A_ currents driven from Cre-positive GABAergic neurons in DAT-cre mice (Straub et al., 2014).

Extracellular dopamine concentration was measured using FCV with 7 μm-diameter carbon fiber microelectrodes (CFMs; tip length 50-100 μm) and a Millar voltammeter (Julian Millar, Barts and the London School of Medicine and Dentistry) as previously (Threlfell et al., 2012). The voltage was applied as a triangular waveform (−0.7 to +1.3 V range versus Ag/AgCl) at a scan rate of 800 V/s and data were sampled at 8 Hz.

For optogenetic stimulation of dopamine release, ChR2-expressing dopamine fibers were activated using a 473 nm diode laser (DL-473, Rapp Optoelectronic) coupled to the microscope with a fiber optic cable (200 μm multimode, NA 0.22). Spot illumination had a 30 μm diameter under × 40 immersion objective. Laser pulses (2 ms duration, 5 pulses at 25 Hz, 23 mW/mm^2^ at specimen) were delivered to mimic physiological firing frequencies and were sufficient to drive dopamine release even with 1 pulse alone.

#### Immunocytochemistry

To verify that recorded neurons were ChIs or SPNs, neurons were filled with neurobiotin. In addition, ChIs were subsequently co-labeled for ChAT as previously (Threlfell et al., 2012). Acute striatal slices were fixed at the end of recordings in 4% paraformaldehyde dissolved in PBS containing 0.2% picric acid. Slices were fixed overnight at 4°C and then stored in PBS. Free-floating sections were then washed in PBS 5 × 5 min and incubated in 0.5% Triton X-100 and 10% normal donkey serum. Slices were subsequently incubated with goat anti-ChAT 1:100 (Millipore) antibody dissolved in PBS containing 0.5% Triton X-100 and 3% normal donkey serum overnight. Sections were then washed with PBS 5 × 5 min and incubated for 2 hr at room temperature with 1:1000 Alexa Fluor 568 donkey anti-goat (Invitrogen) antibody dissolved in PBS containing 0.5% Triton X-100 and 3% normal donkey serum. Alexa 488-conjugated streptavidin (Invitrogen) was included in the secondary antibody solution at a final concentration of 1:250 to identify the recorded neurons. Sections were washed with PBS and mounted on gelled slides with Vectashield mounting medium (Vector Labs) and imaged using an AxioSkop fluorescent microscope (Zeiss).

#### Drugs

Neurobiotin tracer was purchased from Vector Laboratories. Tetrodotoxin (TTX), bicuculline, Zd7288, 4-Aminopyridine (4-AP), and riluzole hydrochloride were purchased from Tocris Bioscience (UK). XE-991 was purchased from Alomone Labs (Israel) and Sigma Aldrich (UK). All all other chemicals were purchased from Sigma Aldrich (UK). Pharmacological drugs were prepared in distilled de-ionized water or DMSO (Riluzole hydrochloride, XE-991 and bicuculline) as stock aliquots at 1000x final concentrations and stored at −20°C. Drug stocks were then diluted to final concentration in carbogenated aCSF immediately before use and were bath-applied.

### Quantification and Statistical Analysis

Statistical analyses used GraphPad Prism 6.0. Data are expressed as mean ± standard error of the mean (SEM). The n value is the number of different neurons. Drug data were normalized to control data before collating across experiments. One-sample t test, t test, Pearson correlations, and paired t test were used.

The amplitude and duration of the pause and rebound illustrated in Figures 1C and 1D were calculated with a MATLAB script. The amplitudes of the pause or rebound were defined from the minimum and maximum value respectively of a moving average (calculated from three 20 ms bins) compared to baseline. The onset or end times of the pause or rebound was defined as the time when a moving average (three 20 ms bins) crossed the baseline level (100%) in normalized data. Correlation between ChI membrane potential and firing rate in Figure 2C used n = 100 bins.

### Data and Software Availability

A single compartment model was run in NEURON (version 7.3; https://www.neuron.yale.edu/neuron). Scripts for our Neuron model have been deposited at https://github.com/Yanfeng-Zhang/Pause-in-ChIs-Neuron-model. The diameter and length of the compartment were set at 15 μm and 40 μm respectively; membrane capacitance was 1 μF/cm^2^; temperature 33°C. This model contained passive leak conductance, an I_A_-conductance, I_Kr_-conductance (Wang et al., 1996) and a current input. The passive leak had a conductance of 0.09 mS × cm^−2^. The resting membrane potential was set to −40 mV to mimic the I_h_ / HCN channel effect on ChIs in normal conditions (Wilson and Goldberg, 2006), and to −76 mV to mimic the condition of blocking I_h_ channel on ChIs (Figure 4D). The I_A_-conductance and I_Kr_-conductances and kinetic parameters, i.e., half activation and inactivation voltage and slope, were taken from experimental data in striatal ChIs (Song et al., 1998). The maximum conductance of I_A_ and I_Kr_ were 2 mS × cm^−2^ and 0.5 mS × cm^−2^ respectively, and the reversal potentials were −85 mV. The synaptic input was mimicked using the current injection feature in NEURON. The evoked D_2_ current in ChIs was modeled using the values for peak and latency shown previously (Straub et al., 2014). A sine shape was used to model the rising of the D_2_ current in Figures 4F–4H, to fit with the rise of phasic activity in DA neuron *in vivo* (Morris et al., 2004). The peak latency and duration of pause response in ChIs and phasic activities in dopamine neurons was simulated using time-courses observed *in vivo* (Morris et al., 2004).

## Supplemental Information

Document S1. Figures S1–S4

Document S2. Article plus Supplemental Information
