# Supplementary material for: Pauses in Cholinergic Interneuron Activity Are Driven by Excitatory Input and Delayed Rectification, with Dopamine Modulation
Source: Neuron. 2018 Jun 6;98(5):918–925.e3. doi: 10.1016/j.neuron.2018.04.027 (PMC5993868; doi:10.1016/j.neuron.2018.04.027)
Supplement: Document S1. Figures S1–S4 [file mmc1.pdf]

Neuron, Volume *98*

Supplemental Information

Pauses in Cholinergic Interneuron Activity Are Driven by Excitatory Input and Delayed Rectification, with Dopamine Modulation

Yan-Feng Zhang, John N.J. Reynolds, and Stephanie J. Cragg

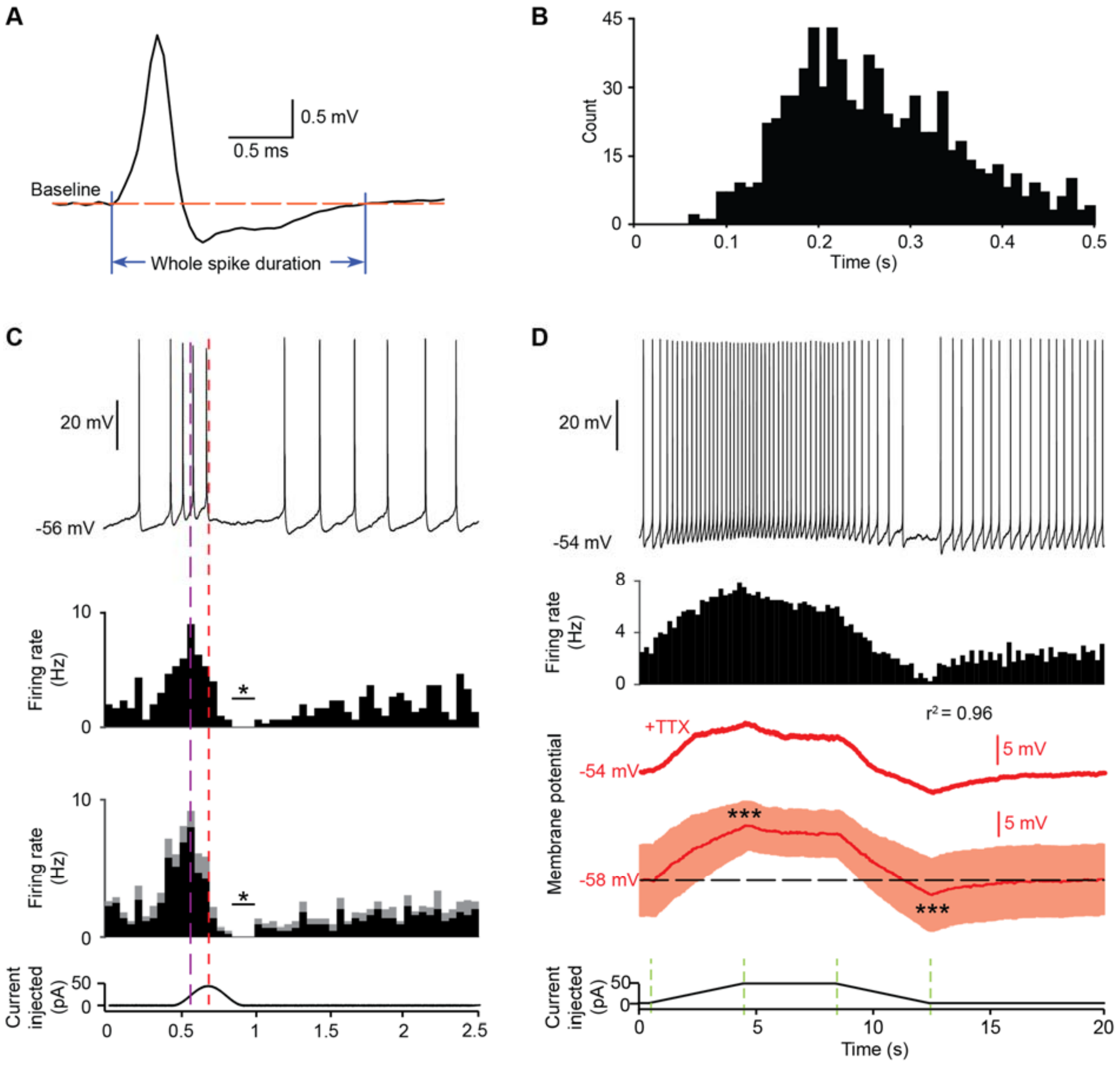

Figure S1
Zhang et al

Figure S1. **Rat ChI firing rate reflects changes to excitatory input. Related to Figures 1 & 2.**
(**A**) A typical characteristic waveform of a pChI recorded *in vivo*, with long whole spike duration. (**B**) Typical firing rate of a pChI *in vivo* is regular with a modal interspike interval (*x*-axis) of ~0.2 s. Neurons were classified as pChIs by tonic firing rate and waveform duration. (**C**) *Upper to lower,* Example sweep, histogram (10 sweeps) and mean histogram ± SEM (*grey shaded*) (*n*=5) of ChI response to sine-shape current. Highest firing rate (*purple dashed line*), input current maximum (*red dashed line*), pause in firing rate versus baseline *P<0.05, t-test. (**D**) Responses to trapezoid current injections for depolarizing input. *Upper to lower*, Example sweep, histogram (10 sweeps), representative and mean (± SEM, *shaded*) membrane potential in TTX 1 μM (*red*), and current injection. Correlation, firing rate and membrane potential, r^2=0.96, 100 bins. ***P<0.001, paired t-test for peaks versus plateaus (*n*=10).

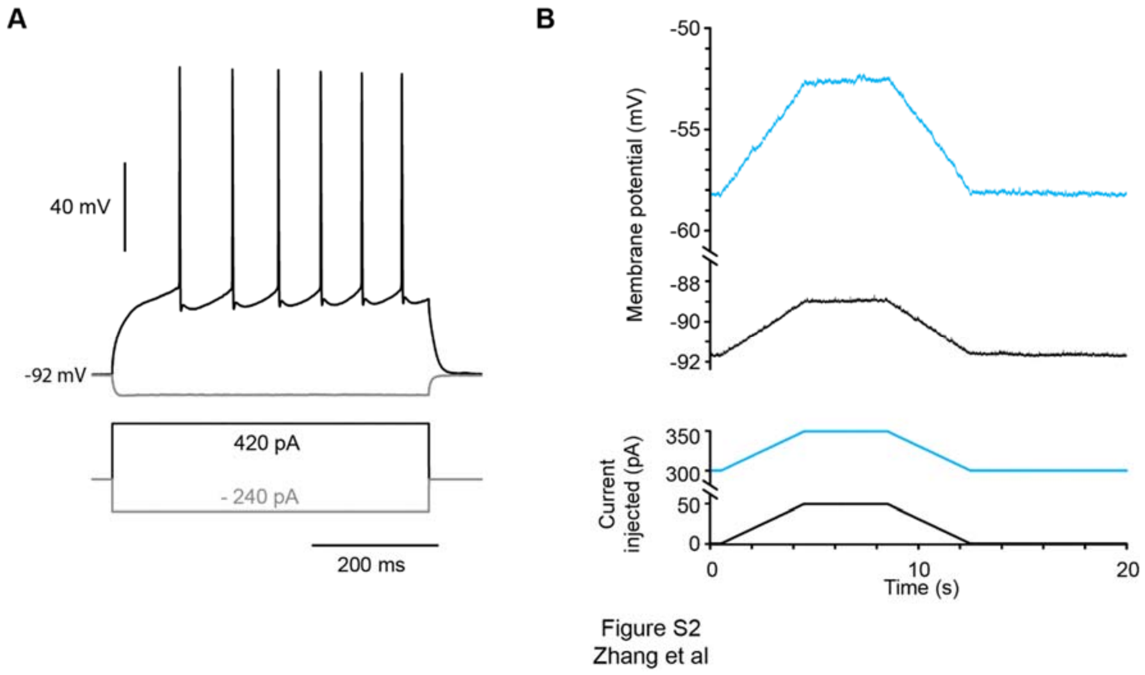

Figure S2. Response of SPNs *ex vivo* to current injection. Related to Figure 2.
(**A**) Characteristic SPN response to depolarizing and hyperpolarizing current injections in acute slices of mouse striatum, including hyperpolarized resting membrane potential and delayed spike activity in response to positive current injection. (**B**) Membrane potential in SPNs responds linearly to current injection when resting membrane potential was normal (-92 mV) or is raised by a holding current to a value (-58 mV, 300 pA holding current, *blue*) that is close to that of ChIs. Representative data, from *n*=5.

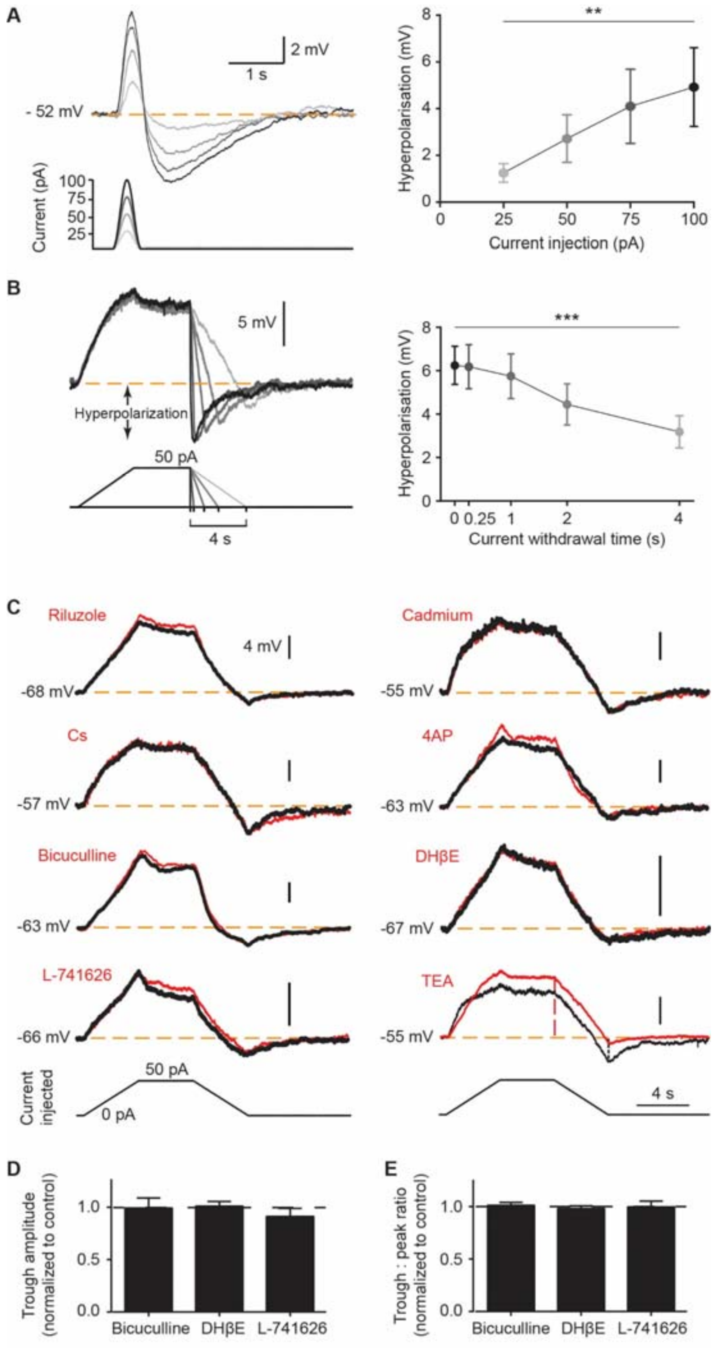

Figure S3
Zhang et al

Figure S3. Hyperpolarization in ChIs scales with amplitude and rate of withdrawal of excitatory input, is TEA-sensitive, and does not require $GABA_A$, nACh or D_2-receptors. Relates to Figures 2 and 3.

(**A**) Typical responses (*left*) of ChI membrane potential (*upper*) to four different amplitudes of sine-shape current injections (*lower*) in ChIs recorded in striatal slices. Hyperpolarization (± SEM) in ChIs following input withdrawal scales with depolarization (*right*, *n*=5, ***P*<0.01, Pearson's correlation). (**B**) Typical responses (*left*) of ChI membrane potential (*upper*) to withdrawal from depolarizing current of 50 pA to 0 pA at different rates. Hyperpolarization trough amplitude (± SEM) varies inversely with duration of current withdrawal, (*right, n*=5, ****P*<0.001, One-way ANOVA). (**C**) Typical recordings of before (*black*) and during (*red*) either riluzole, cadmium, CsCl, 4-AP, bicuculline, DHβE, L-741626 or TEA. Resting membrane potentials were kept at pre-drug values using current injections when necessary. Vertical scale bar, 4 mV. (**D**) Mean ± SEM of amplitude of hyperpolarization trough (*black dashed*) from RMP (*orange dashed*) or (**E**) mean ± SEM of ratio of hyperpolarization trough to peak (*black* versus *red dashed lines* in *panel C*), normalized to control condition for bicuculline 10 μM, DHβE 1 μM, L-741626 1 μM.

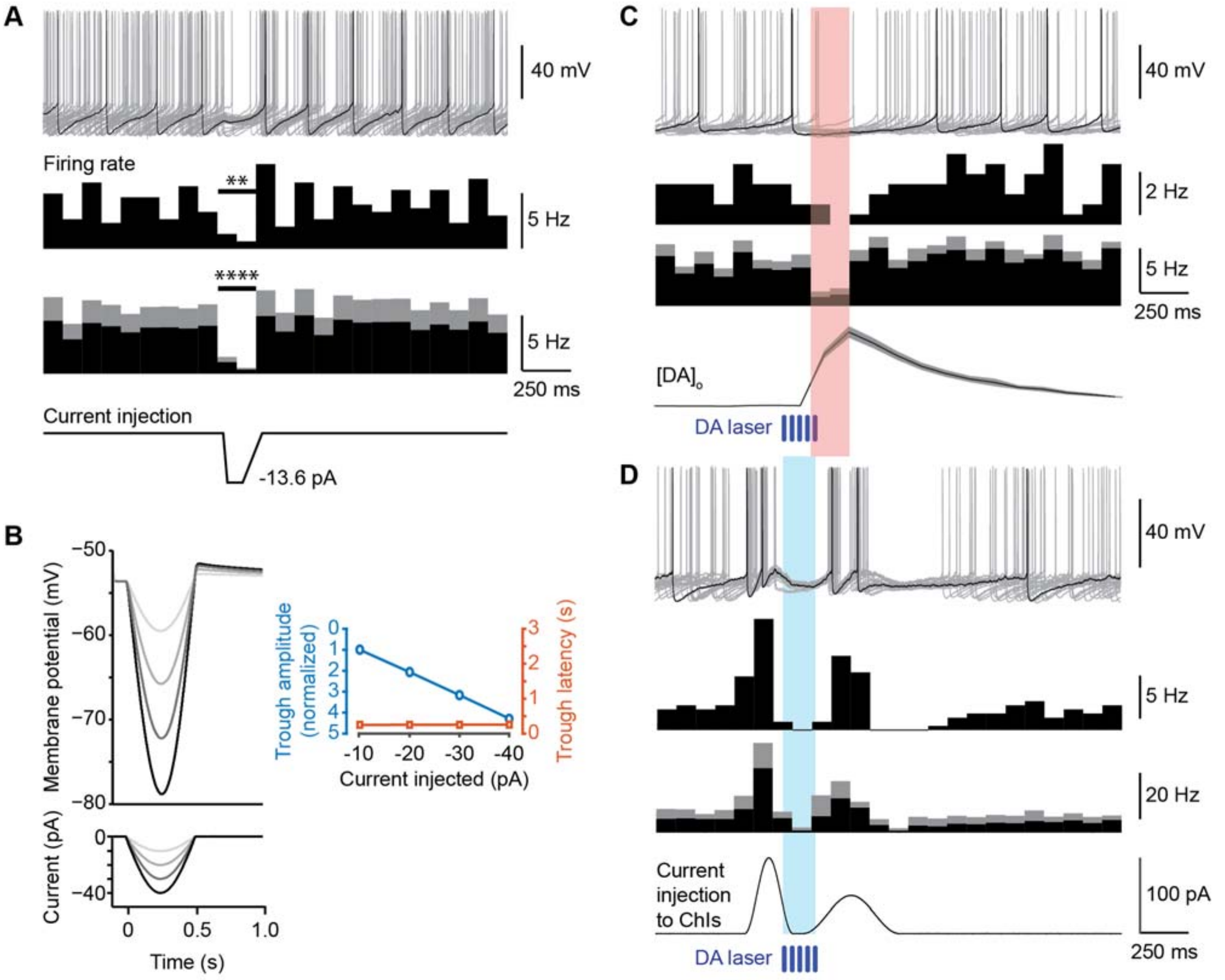

Figure S4
Zhang et al

Figure S4. ChIs can pause in response to changing input current or burst activity in dopamine neurons, but when concurrent, dopamine effects are too slow. Relates to Figure 2 and 4.
(**A**) Firing rate in mouse ChI in slices showing (*upper to lower): e*xample activity (20 sweeps), example firing rate histogram, mean firing rate histogram ± SEM (*n*=5), in response to -13.6 pA current injection (equivalent to the I_{Kr} current evoked by a 50 pA sine wave, see Methods). ***P*<0.01, *****P*< 0.0001, t-test. (**B**) Response of ChI membrane potential (*upper*) to a series of sine-shape hyperpolarizing current injections (*middle*) in the computational model shown in Fig. 4. *Right*, the trough amplitude (*blue*) but not latency (*orange*) scales with amplitude of current injected. (**C**) Firing rate in mouse ChI recorded in slices showing (*upper to lower*): example activity (15 sweeps), example firing rate histogram, mean firing rate histogram ± SEM (*n*=5), as well as extracellular dopamine concentration ($[DA]_o$) (mean ± SEM) detected with voltammetry (*n*=14), in response to optogenetic activation of dopamine axons with blue light pulses (*DA laser*). Dopamine-induced pause, *red shade.* (**D**) Firing rate in mouse ChI recorded in slices showing (*upper to lower*): example activity (20 sweeps), example firing rate histogram, mean firing rate histogram ± SEM (*n*=5) of ChI response to current injection, while concurrently dopamine axons were light-activated *(DA laser,* 5 pulses) to coincide with the pause induced by withdrawal of excitation of ChIs (*blue shade*). The dopamine-induced pause seen in C occurs too late to coincide with the ChI pause in D.
